# Development and implementation of a strategy for early diagnosis and management of scrub typhus: an emerging public health threat

**DOI:** 10.3389/fpubh.2024.1347183

**Published:** 2024-04-10

**Authors:** Hitesh Kumar Jain, Arundhuti Das, Sujata Dixit, Harpreet Kaur, Sanghamitra Pati, Manoranjan Ranjit, Ambarish Dutta, Madhusmita Bal

**Affiliations:** ^1^Department of Molecular Epidemiology, Indian Council of Medical Research, Regional Medical Research Centre, Bhubaneswar, India; ^2^Department of Epidemiology and Communicable Diseases, Indian Council of Medical Research, New Delhi, India; ^3^Department of Public Health, Indian Council of Medical Research, Regional Medical Research Centre, Bhubaneswar, India; ^4^Department of Epidemiology, Public Health Foundation of India, Indian Institute of Public Health, Bhubaneswar, India

**Keywords:** scrub typhus, implementation research, protocol, diagnosis, disease management, public health, control program

## Abstract

Scrub typhus, caused by *Orientia tsutsugamushi*, is a re-emerging zoonotic disease in the tropics with considerable morbidity and mortality rates. This disease, which is mostly prevalent in rural areas, remains underdiagnosed and underreported because of the low index of suspicion and non-specific clinical presentation. Limited access to healthcare, diagnostics, and treatment in rural settings further makes it challenging to distinguish it from other febrile illnesses. While easily treatable, improper treatment leads to severe forms of the disease and even death. As there is no existing public health program to address scrub typhus in India, there is an urgent need to design a program and test its effectiveness for control and management of the disease. With this backdrop, this implementation research protocol has been developed for a trial in few of the endemic “pockets” of Odisha, an eastern Indian state that can be scalable to other endemic areas of the country, if found effective. The main goal of the proposed project is to include scrub typhus as a differential diagnosis of fever cases in every tier of the public health system, starting from the community level to the health system, for the early diagnosis among suspected cases and to ensure that individuals receive complete treatment. The current study aimed to describe the protocol of the proposed Scrub Typhus Control Program (STCP) in detail so that it can receive valuable views from peers which can further strengthen the attempt.

## 1 Introduction

### 1.1 Scrub typhus disease, its diagnosis, and its treatment

Scrub typhus, also known as bush typhus, is a re-emerging but neglected zoonotic acute febrile illness. Approximately 1 million people worldwide are affected by this disease annually, with a mortality rate of nearly 50% if untreated, and 1 billion people are at risk of infection globally (1, 2). This disease is caused by *Orientia tsutsugamushi*, an obligate intracellular gram-negative *Coccobacillus* belonging to the family Rickettsiae. Rodents are the natural reservoir, while trombiculid mites (“chiggers,” *Leptotrombidium deliense*, and others) act as a vector as well as a reservoir of the causative bacteria. Scrub typhus is transmitted to humans by the bite of infected chiggers (larvae) of trombiculid mites (3, 4).

The clinical manifestations of scrub typhus range from a mild and self-limiting illness to severe or even fatal illness. The initial clinical signs include an eschar, indicating localized skin necrosis where the mite has fed (though it may not always be present), along with nearby lymph node, followed subsequently by fever, headache, myalgia, generalized lymphadenopathy, cough, gastrointestinal symptoms, transient hearing loss, and rash (5, 6). However, there are wide variations in the clinical manifestations of the disease, and the reasons for such variations are mostly unknown. Severe scrub typhus manifests as acute respiratory distress, meningoencephalitis, gastrointestinal bleeding, acute renal failure, hypotensive shock, and coagulopathy (7). Studies from Assam have reported that *Orientia tsutsugamushi* infection can lead to acute encephalitis syndrome (AES) and subsequent death (8). Occasionally, the case fatality can be as high as 30%−70% in untreated cases (9, 10). Furthermore, the wide genetic variability observed in the pathogen in India, such as Gillam, Karp, Hualien1, and Keto, and across the globe, along with complicated host–pathogen interactions, hinders the development of vaccine and improved diagnostic and therapeutic methods (8, 11–14).

The identification of an eschar at the site of mite bites serves as a highly specific (98.9%) indicator for diagnosing scrub typhus (15). However, the presence of an eschar in Indian and other Asian populations is rare, which makes it an inappropriate method for the diagnosis of scrub typhus. Therefore, the diagnosis predominantly depends on laboratory tests (11). Serological assays such as the Weil–Felix test, indirect immunofluorescence assays, indirect immune peroxidase assays, and enzyme-linked immunosorbent assay (ELISA) and immune chromatographic tests (ICTs) are the prominent tests employed to diagnose rickettsial diseases. Among all serological assays, the IgM ELISA-based method is the most reliable one for the diagnosis of scrub typhus (16). Compared to other tests, the Weil–Felix test lacks high sensitivity and specificity and can serve as a useful and inexpensive primary diagnostic tool that can be performed by laboratory technicians at peripheral hospitals (17, 18). Other assays are not widely recommended in primary care. The Indian Council of Medical Research (ICMR) has recently developed guidelines outlining the diagnosis and treatment of rickettsial diseases. These guidelines include presenting manifestations, case definitions, laboratory criteria (both specific and supportive investigations), and recommended treatments (17).

The drug of choice for the treatment of diseases within the order Rickettsiales is doxycycline, while tetracycline, chloramphenicol, and azithromycin have also demonstrated efficacy (19). However, there is growing concern about the development of antibiotic-resistant strains of *O. tsutsugamushi*, both at present and in the future.

### 1.2 Epidemiology of scrub typhus

#### 1.2.1 Global

The areas where the disease is prevalent is known as the “Tsutsugamushi Triangle,” encompassing Japan in the east, Afghanistan and the Middle East in the west, and various regions in between such as the Pacific Islands, North Australia, Indonesia, Southeast Asia, China, and Korea. The Tsutsugamushi triangle is home to more than half of the world's population, with 2 billion people at risk and 1 million cases of scrub typhus occurring per year (20).

Nevertheless, scrub typhus is currently increasingly identified in regions where the disease was previously unfamiliar or had been largely overlooked, including India, Sri Lanka, the Maldives, and Micronesia. Recently, scrub typhus has been reported beyond the boundaries of the Tsutsugamushi triangle, causing concerns across the globe about the pathogen (4, 21).

#### 1.2.2 India

Scrub typhus is a serious public health problem in India causing severe morbidity and a significant number of deaths (22). It has been reported in different geographical zones of India: Kerala, Karnataka, Andhra Pradesh, and Tamil Nadu in South India; Bihar, Maharashtra, Jammu Kashmir, Himachal Pradesh, Uttaranchal, and Rajasthan in Northern India; and Meghalaya, Sikkim, and West Bengal in Northeast India (6, 23–28).

Evidence has been emerging, although mostly from small-scale regional studies, that scrub typhus is prevalent mainly in many rural areas of India, especially among vulnerable populations such as poor farmers, children, older adults, and pregnant women. In some parts of India, this prevalence has resulted in the characterization of scrub typhus as a re-emerging infectious disease in India, emphasizing a critical public health concern for this illness in the country (8, 29). In some settings in India, ~20%−24% of all patients presenting with unexplained febrile illness has been reported to be due to scrub typhus, and 53% of patients have evidence of acute kidney injury (30–32). A study showed that the circulating genotypes of *O. tsutsugamushi* in three scrub typhus–endemic geographic regions of India: South India, Northern India, and Northeast India—provide potential resources for future region-specific diagnostic studies and vaccine development (29).

#### 1.2.3 Odisha

Odisha is located within the endemic belt of scrub typhus, but the disease continues to remain unrecognized, underdiagnosed, and underreported. Only recently, few reports from a private tertiary care hospital in the capital city confirmed many cases of undifferentiated fever as scrub typhus (33–35). The authors of these reports observed that scrub typhus patients were hospitalized due to either prolonged fever (more than 10–30 days) or complications such as encephalopathy/AES, acute renal failure, acute respiratory distress, and multiorgan failure cases with unknown etiology. Among children, AES/encephalopathy was the most common cause of hospitalization followed by hepatic involvement, shock, and renal failure.

However, none of the studies cited above were conclusive about the population burden of scrub typhus, even from any particular district or “pocket” in the state, because all of these studies were conducted in tertiary health facilities, and the cases would be only those with extreme severity requiring hospitalization and those who would have presented from all over the state. However, many cases from the Keonjhar district were found to be positive for scrub typhus by molecular tests conducted at the reference laboratory of the state, The Regional Medical Research Center (RMRC), Bhubaneswar, an institute of ICMR, which is because few physicians from the district clinically suspected scrub typhus in many prolonged fever cases that presented to them and referred them for molecular testing at RMRC. In the backdrop of these two observations, first from the tertiary hospital in the capital city and the second from the primary care setting in Keonjhar, an exploratory study was undertaken by the RMRC to examine the prevalence of scrub typhus in few suspected high-endemic pockets of Keonjhar. The study showed high prevalence (39.7%) of scrub typhus among children with prolonged fever during July–September, conventionally the peak transmission season for other diseases such as dengue, malaria, and other viral diseases. Among them, eschar was found in 17.9% of the cases (36). Meanwhile, the serological analysis undertaken by RMRC of 30 AES archived samples collected from hospitalized children during the 2016 epidemic from another district, Malkangiri, indicated that 23.3% (7/30) of the cases were positive for scrub typhus. Recently, high incidence of scrub typhus associated with acute kidney injury was reported among patients having a history of acute fever with various degrees of kidney involvement admitted to a tertiary care hospital (11).

### 1.3 Rationale and aims

Despite its high estimated burden, the WHO has described scrub typhus as one of the most underdiagnosed and underreported febrile illnesses requiring hospitalization. It strongly emphasizes surveillance owing to its relatively high fatality rate (up to 30% in untreated patients).

It has been suggested that deaths from scrub typhus may exceed those of dengue fever globally. However, dengue in India and elsewhere still attracts far higher public and professional awareness, whereas scrub typhus is outside the “limelight” (6). A recent editorial in the *New England Journal of Medicine* described scrub typhus as “probably the single most prevalent, under-recognized, neglected, and severe but easily treatable disease in the world” (37).

Although scrub typhus is currently increasingly found in metropolitan areas, it still remains predominantly a disease affecting the rural population of low and middle income countries (LMICs) that includes India (38). The rural populations in such settings are often mired with limited access to healthcare, diagnostics, and treatment, which further makes it difficult to differentiate scrub typhus from other febrile illnesses on a clinical basis alone. Consequently, scrub typhus tends to be largely ignored, especially amid fever outbreaks, and its progression often becomes complex, resulting in severe complications and even fatalities without proper intervention. Nonetheless, with early diagnosis and the prompt initiation of specific treatment, the chances of cure rates are significantly high (39).

As solid evidence of a considerable burden of scrub typhus in Odisha emerged from reports of tertiary care hospitals and the community-based study of the RMRC (11, 36), there was a necessity to initiate a public health program that can be instituted to control this problem in the state so that many severe health conditions and fatalities related to scrub typhus at the population level can be averted in the long term.

Therefore, the RMRC proposed to conduct an implementation research project (hereinafter referred to as the research project), the cornerstone of which would be designing and rolling-out a replicable and scalable template of Scrub Typhus Control Program (STCP) within the public health system of the state. The research project will also thereafter entail the assessment of the effectiveness of STCP. The current study aims to describe the protocol of the research project in detail so that it can receive valuable suggestions from peers, which can further strengthen the endeavor.

## 2 Methods and results

The research project has two distinct sections and will entail the following broad areas of activities, with each area of activity having its own set of objectives.

- STCP design and its roll-out
  · Empiric selection of the study site where the STCP will be rolled-out and which area will be considered as control unit for evaluation.
  · Preparation for STCP roll-out
  · STCP roll-out
- Evaluation of the STCP

### 2.1 An outline of the public health system of Indian states

**Figure 1:**
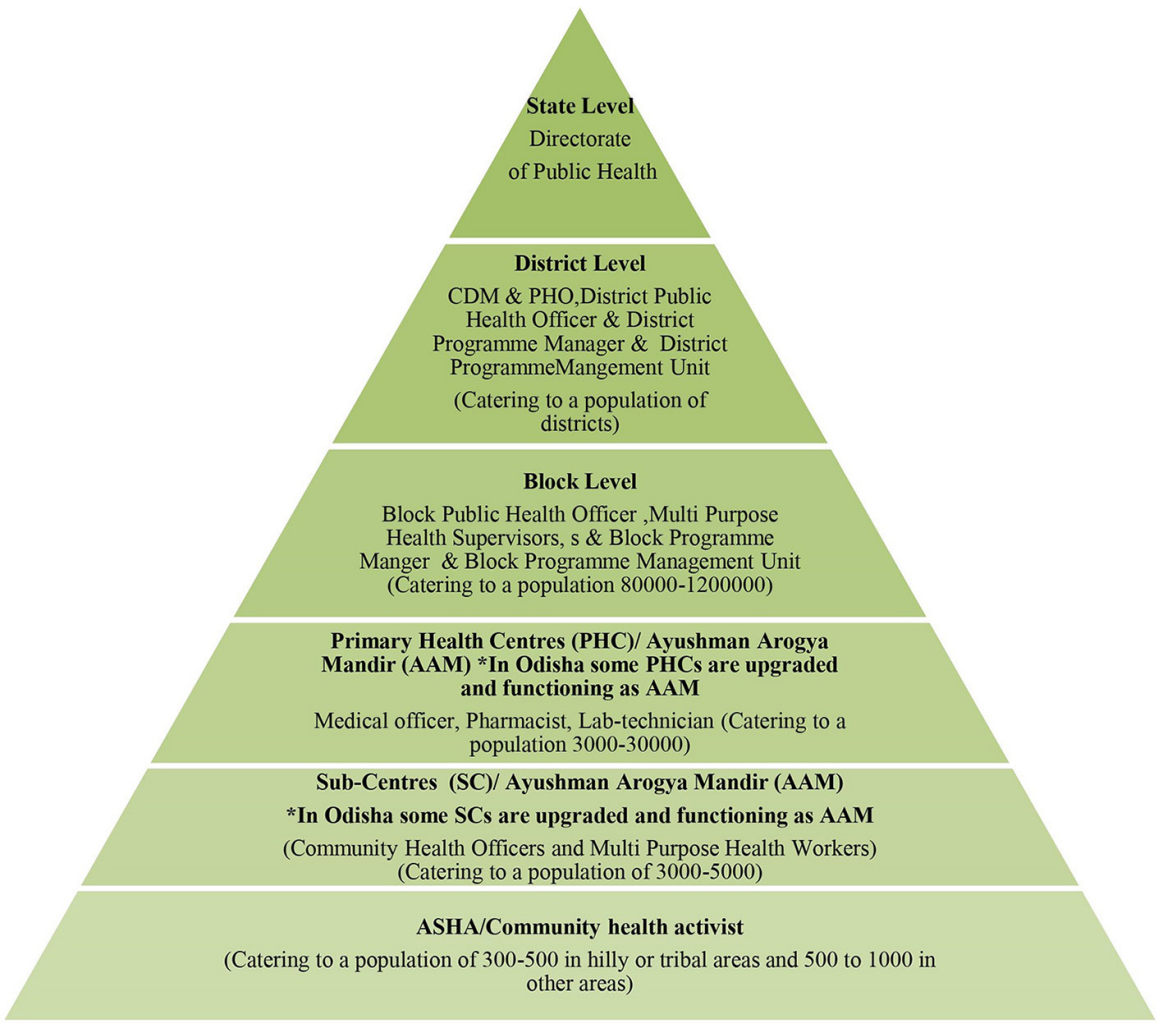
Hierarchy of the public health system in India.

The proposed STCP will be rolled-out entirely through the existing public health system of Indian states (Figure 1) without any additional resources, except the provision of logistics to equip public health laboratories for diagnosis of the disease under the designed program so that it becomes a sustainable model.

### 2.2 Description of scrub typhus control program (STCP)

#### 2.2.1 Conceptual framework guiding the design of STCP

**Figure 2:**
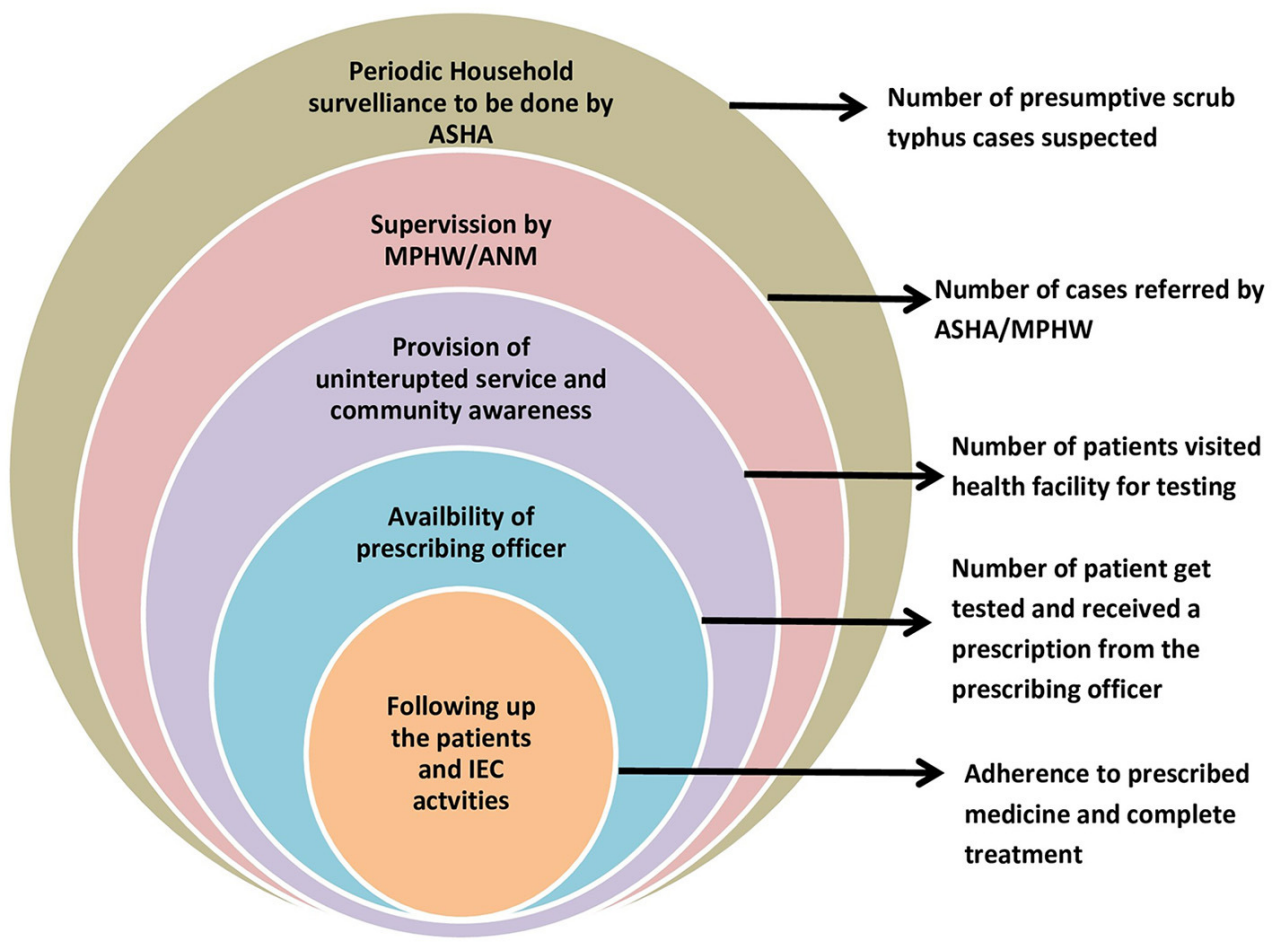
Conceptual framework for studying the continuum of care for scrub typhus across the health system.

A commonly used conceptual framework, often referred to as the cascade of care, in exploring the control of diseases such as tuberculosis and hypertension was customized for designing and planning a public health program to control scrub typhus—STCP. The conceptual framework is graphically presented below (Figure 2), which comprises different steps of scrub typhus control, starting from suspecting scrub typhus in the population to completing the treatment of confirmed cases in health facilities. Other than natural attrition from the care cascade, for example, many suspected cases turn out to be not diseased after testing and hence they drop out of the program. There are certain programmatic “losses” envisaged through this framework, thus not all suspects may be identified at the community level and all identified suspects may not undergo screening/diagnosis. To summarize, it was presumed from anecdotal experiences that the suspicion index for scrub typhus among all categories of health workers, including physicians, was low and that diagnostic tests for this disease was not available in primary care (11, 36). Therefore, these two components were identified as “rate limiting steps” for scrub typhus control and guided the design of the STCP so that the potential losses from the care cascade can be preemptively plugged and program effectiveness optimized.

#### 2.2.2 Objective of STCP

Based on the conceptual framework mentioned above, the objectives of the proposed STCP are as follows:

- Scrub typhus to be included as a differential diagnosis of fever cases in every tier of the public health system
- Screening and diagnosis among presumptive scrub typhus cases to be enhanced
- Complete treatment of diagnosed scrub typhus cases to be ensured.

#### 2.2.3 Main activities of STCP

**Figure 3:**
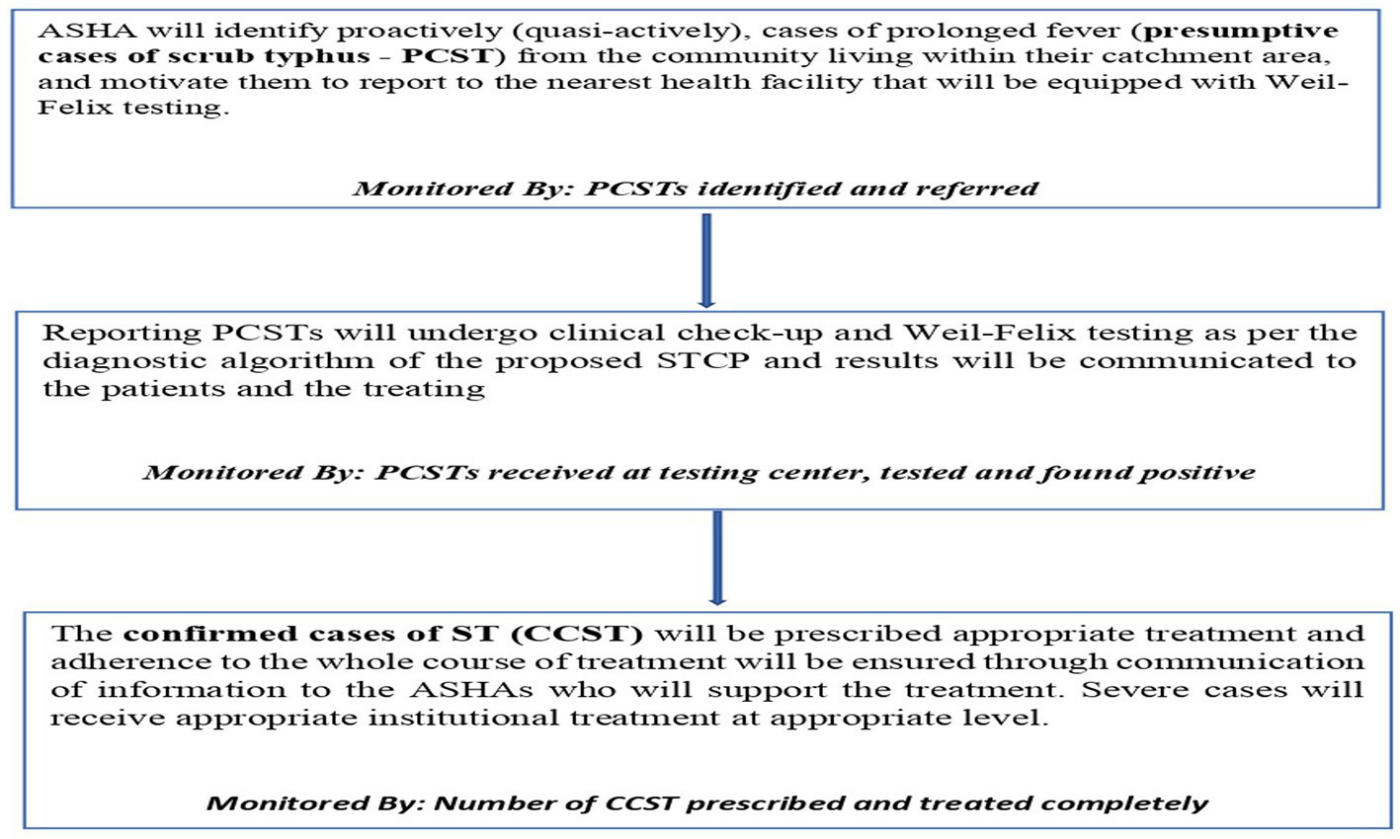
Flowchart showing the main activities of the STCP.

Based on the objectives of the STCP, the main activities to be carried out by the STCP along with their monitoring indicators are shown in Figure 3.

### 2.3 Diagnosis and treatment algorithm of the STCP

**Figure 4:**
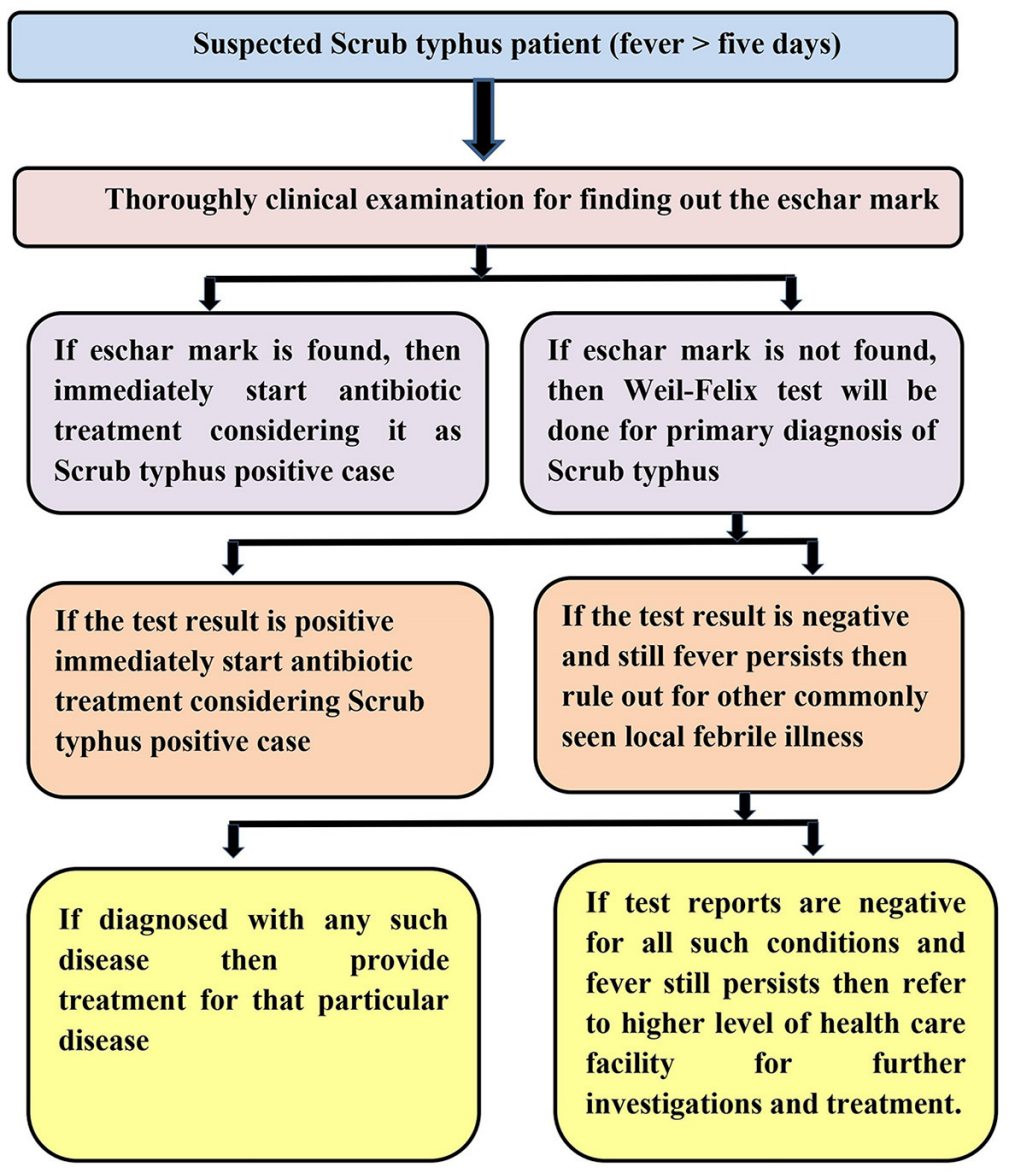
Flowchart showing the diagnostic algorithm of the STCP.

A diagnostic algorithm has already been developed in line with the ICMR guidelines for treating scrub typhus but is customized to the local context and STCP objectives. This algorithm will be applied to diagnose and treat cases of scrub typhus in the proposed program (Figure 4).

### 2.4 Outline of research project

Herein, we delineate four broad areas of activities (with their respective objectives and micro-actions mapped to them (Table 1) for the proposed implementation research project:

**Table 1:** Broad area of activities, purposes, and the corresponding micro-plans of the implementation research project.

**Broad areas of activities**	**Purpose**	**Micro-plan of actions to be taken**
Empiric selection of study sites	To select the site where the proposed program will be implemented and subsequently evaluated	• Collection of secondary data for prolonged fever for last 3 years from the Integrated Disease Surveillance Program (IDSP) cell and its analysis
Preparation for STCP roll-out	To ensure preparatory activities for seamless roll-out of the STCP at the intervention site(s)	• Advocacy meetings and stakeholder mapping • Development of different training modules for health workers at different levels and IEC materials to educate the community • Development of the STCP information system • Introduction of the Weil-Felix test in the existing public health laboratory network • Setting of a molecular testing facility at the RMRC • Training of frontline health workers for identification of suspects and referrals • Training of lab technicians for the Weil-Felix test • Training of MOs for complete treatment
STCP roll-out	• To roll-out STCP • To monitor whether the implementation is progressing as per plan/design • To roll-out STCP MIS • To ensure quality assured lab services—carry-out molecular research	• IEC in the community level using different mediums in the local language • Monitoring of the referral system and • Supportive supervision • Data collection, extraction, and analysis of STCP information system data • Reorientation or refresher training for health workers • Monthly meeting at the sector level • Quality assurance of lab services • Transportation of samples to the RMRC for molecular tests
Program evaluation	• To estimate the effectiveness of STCP	• Baseline community survey • Health system assessment • End-line survey • Analysis of time-series information system data

The broad areas of activities of the implementation research project are described. Then, we mapped the objectives to them and planned the micro-level actions to be carried out to achieve the objectives. All these three verticals are summarized in the matrix below. Some of the putative activities are elaborated later. The first activity of the STCP has already been carried out, which was essential for the selection of the STCP setting.

Some of the actions mentioned above are elaborated below:

#### 2.4.1 Secondary data analysis and selection of the study site

A secondary data analysis has already been conducted for exploring the burden of scrub typhus in various sub-district units (known as “blocks” in India which represent the lowest administrative unit in Indian states) of the Keonjhar district of Odisha with a view to select the study site. This was planned as a forerunner to the design, implementation and evaluation phase of STCP as mentioned above. Since there is no scrub typhus specific data currently available in the health information system and a previous study by RMRC has confirmed that scrub typhus cases are hidden amid the unidentified fever cases only, only cases of prolonged fever (of 5 days or more) was decided to be considered as presumptive cases of scrub typhus. Year-wise data for the last 3 years on prolonged febrile illness available with Integrated Disease Surveillance Program cell of Odisha, Directorate of Health Services, Govt. of Odisha, and hospital records of all the 15 Community Health Center (CHC) and district headquarter hospital were collected, collated, and analyzed to assess the magnitude and prepare the distribution map. By analyzing the data, four blocks of Keonjhar with highest prolonged fever cases and with >60% tribal population has been selected as the study setting (described below).

**Figure 5:**
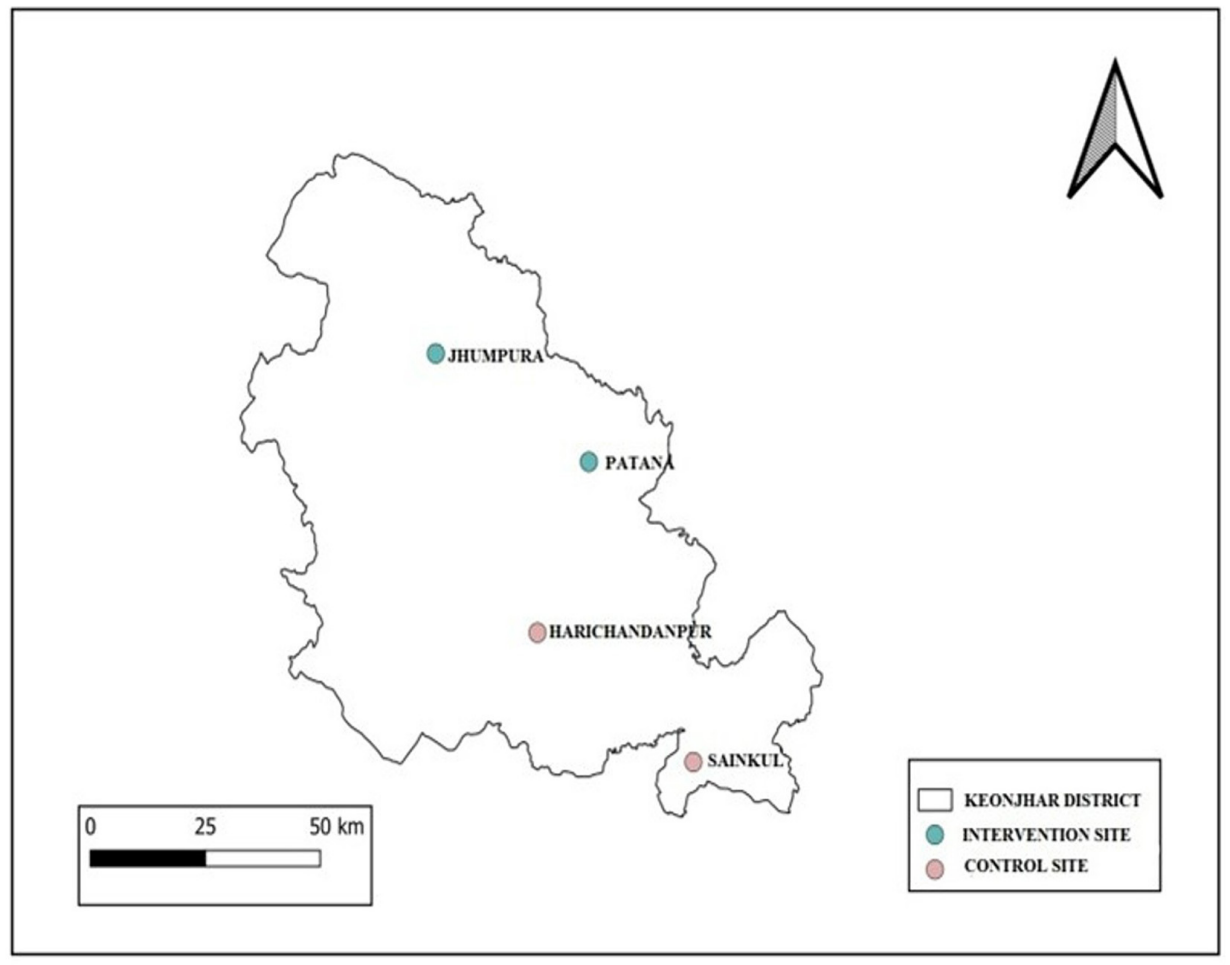
Map showing the study sites.

The total population of the district is 1,801,733(2011 census), of which > 44.5% belongs to tribal communities. There are 59 Primary Health Center (PHC) and 226 sub-centers under 15 CHC, 2 sub-divisional hospitals, and one district headquarter hospital in the districts. The district has been selected based on our preliminary observation that scrub typhus is one of the major cause of unexplained febrile illness among children < 15 years of age (36). Four CHC, two for implementation at Jhumpura and Patana and two for comparison (control) at Harichandanpur and Sainkul CHC, are selected. Allocation of blocks to control and intervention arms were done randomly (Figure 5).

#### 2.4.2 Baseline survey

A baseline survey will be conducted among the patients visiting the outpatient department in selected CHC and PHC under them with complaints of prolonged fever. This survey will provide an overview of scrub typhus burden. Following this survey, a community assessment survey will be conducted using the cross-sectional study design with the objective of finding out the knowledge attitude and practices of people regarding the disease and their health seeking behavior toward febrile illness. Additionally, a health system assessment will be performed to understand the readiness of the existing public health system in implementing the proposed STCP.

#### 2.4.3 IEC and training material/module development

The training modules for healthcare workers will be prepared with the help of experts using the existing literature. A total of four training modules will be prepared for medical officers, laboratory technicians and pharmacists, community health officers (CHOs), multi-purpose health workers (MPHWs), and accredited social health activists (ASHAs). These training modules will contain a brief overview about scrub typhus biology, the burden of the disease, its transmission cycle, signs and symptoms, clinical manifestations, diagnosis, case management, treatment, controlling strategy, primary prevention, protocols for sample collection, and the Weil-Felix test procedure and their role in project implementation.

The Information, Education, Communication (IEC) materials (posters, leaflets, and audio-visuals) will be prepared in local languages to deliver precise, simple, comprehensive and practicable messages. The IEC materials and the communication strategy will be developed through the ‘participatory learning and action' approach by assessing the knowledge, practices, taboos, beliefs, and misconceptions prevalent in the community related to treatment and prevention of the disease.

#### 2.4.4 Training

The healthcare providers at all levels will be trained by organizing district-level sensitization workshops with the help of experienced clinicians, rickettsia experts, and public health experts. Following those training, individual training workshops will be organized at the CHC and sector levels. Regular meetings/workshops will be organized at the field level on epidemiology and public health importance of scrub typhus and clinical features, diagnosis, clinical management, and treatment of those patients. Refresher training sessions will be organized periodically.

#### 2.4.5 IEC

IEC activities, such as street plays called “*Pala*” in the local language, wall paintings, sensitization programs at schools, meetings with villagers at the village health nutrition day, *Village-welfare* meetings, and meetings with mothers on immunization day, will be conducted for sensitizing and creating awareness regarding scrub typhus.

#### 2.4.6 Setting up the laboratory network and quality assurance

The CHCs and primary health centers (now referred to as “health and wellness centers” in the Odisha context) will be established as referral and diagnostic units. These are already equipped with basic logistics for treatment and diagnosis along with manpower, such as community health officers, to facilitate the same. The existing public health laboratory network will be equipped with the Weil-Felix test for the primary diagnosis of scrub typhus at the peripheral level. Laboratory technicians will be trained for conducting the test and result interpretation. For quality assurance of the testing, samples will be shipped to the ICMR-RMRC molecular laboratory via proper cold chain for conducting IgM ELISA followed by PCR.

#### 2.4.7 STCP information system

An information system (STIS) will be developed for the STCP using the forms and registers at different levels of the healthcare system, as described in Table 2, to track the functioning of the referral system. The indicators will be computed using the data extracted from the STIS for the monitoring and evaluation of the STCP.

**Table 2:** Forms, registers, and indicators used at different levels of STIS.

**Level**	**Document**	**Indicators**
ASHA	• Register: The existing minor element register will be used for registering PSTC • Referral form • Monthly reporting format	• Number of PSTC identified • Number of PSTC referred • Number of PSTC receiving tested for scrub typhus • Number of PSTC tested positive for scrub typhus • Number of CCST receiving complete treatment • Number of testing done at the laboratory
Lab	• Lab register • Lab form • Monthly Lab Reporting Format	
Medical Officer	• Treatment form	
Block	• Monthly reporting format	

#### 2.4.8 Supervision and monitoring

Monitoring and supervision of the planned activities under the STCP will be done periodically for ensuring the optimum implementation of the STCP as per the plan. The project team will be attending every meeting of the field staff for discussing the functioning of various components of the programs and trying to find solutions to challenges and barriers. Meetings will be also held periodically with higher-level, sub-district, and district-level officials to discuss the ongoing STCP, the challenges faced, and their possible solutions.

#### 2.4.9 End-line survey

An end-line survey will be conducted in the same population where the baseline survey was conducted using same methods and tools. The findings of this survey will be used during the evaluation of the proposed STCP.

### 2.5 Evaluation

The evaluation will be carried out using a difference-in-difference framework. The framework will use knowledge and practice outcomes of community members and health system personnel from baseline and end line cross-sectional surveys. The evaluation process will analyze the time series of seminal indicators recorded by the STCP information system as follows:

- Number (percentage) of Presumptive Scrub typhus Case (PSTC) suspected.
- Number (percentage) of PSTC referred.
- Number (percentage) of PSTC tested.
- Number (percentage) of CCST.
- Number (percentage) of CCST cases that received complete treatment.

The evaluation will also examine the input and the process involved in the roll-out of the STCP such as training, community engagement, and IEC.

### 2.6 Ethical clearance

The study has been approved by the Institute of Human Ethics Committee (ICMR-RMRCB/IHEC-2020/030) and Research & Ethics Committee, Department of Health and Family Welfare, Government of Odisha [22516/MS-2-IV-04-2020 (PT-1) Dated 23/11/2021].

## 3 Discussion

Recent recognition of the substantial burden of scrub typhus in the South Asian region, including India, can be ascribed not only to increased reporting of the infectious disease but also to perhaps increasing incidence of the infection due to the ongoing social, environmental, and economic changes, which has left the region more vulnerable to such zoonotic diseases. Human activities, such as clearing of forest, aggressive agricultural practices, urbanization, continued poor sanitization, and close proximity of people to livestock, along with climate change, are accelerating human interaction with natural hosts of such diseases. The ‘One Health' approach, which advocates the transdisciplinary collaborative relationship between humans, animals, and environment health partners to address the emergence and re-emergence of the zoonotic disease, is unquestionably the best possible strategy for overall control of scrub typhus (40, 41). The current proposed program described in this protocol, however, is yet to adopt the ‘One Health' approach. The proposed program primarily focuses on establishing the structures for Early Diagnosis and Complete Treatment (EDCT) for reducing severe morbidity and mortality rates caused by scrub typhus. The premises for this strategy is that, once this basic EDCT infrastructure is well established and functioning optimally, in the subsequent phases of the project, the endeavor should be to include the control of non-human components of the disease for its wholistic control, namely, animal host of the disease and environment, by employing “One Health” and climate-adaptive approaches. The proposed study is the first of its kind in the country which entails designing, roll-out, and assessment of a scrub typhus control program from a tribal district of Odisha. The choice of a district, which is remote and challenging, infrastructure-wise and designing the project to run entirely through the existing local public health system ensure its sustainability, replicability, and scalability in any part of the country where it is needed.

## Ethics statement

The studies involving humans were approved by Institute Human Ethical Committee (ICMR-RMRCB/IHEC-2020/030) and Research Ethics Committee, Department of Health and Family Welfare, Government of Odisha [22516/MS-2-IV-04-2020 (PT-1) Dated 23/11/2021]. The studies were conducted in accordance with the local legislation and institutional requirements. Written informed consent for participation in this study was provided by the participants' legal guardians/next of kin.

## Author contributions

HJ: Data curation, Investigation, Methodology, Supervision, Writing—original draft, Visualization. ADa: Investigation, Methodology, Writing—review & editing. SD: Writing—review & editing, Data curation, Investigation. HK: Writing—review & editing, Project administration, Funding acquisition, Resources. SP: Funding acquisition, Project administration, Supervision, Writing—review & editing. MR: Conceptualization, Funding acquisition, Investigation, Project administration, Writing—original draft, Writing—review & editing, Methodology, Resources, Supervision, Visualization. ADu: Conceptualization, Investigation, Methodology, Project administration, Writing—original draft, Writing—review & editing, Visualization. MB: Investigation, Methodology, Conceptualization, Funding acquisition, Project administration, Resources, Supervision, Visualization, Writing—original draft, Writing—review & editing.
